# Infection Prevention and Control Challenges With First Pregnant Woman Diagnosed With COVID-19: A Case Report in Al Ahssa, Saudi Arabia

**DOI:** 10.7759/cureus.10035

**Published:** 2020-08-26

**Authors:** Ahmed AlOmran, Yameen Almatawah, Bushra Al Sharit, Zainab Alsadah, Ola Mousa

**Affiliations:** 1 Infection Control, Maternity and Children Hospital, Al-Ahsa, SAU; 2 Pediatric Infectious Disease, Maternity and Children Hospital, Al-Ahsa, SAU; 3 Maternity and Children, Maternity and Children Hospital, Al-Ahsa, SAU; 4 Nursing, King Faisal University, College of Applied Medical Sciences, Al-Ahsa, SAU

**Keywords:** pregnancy with covid 19, infection prevention and control

## Abstract

This study report focuses on facts on a pregnant woman of COVID-19 who admitted to Al Ahsa Maternity and Children Hospital on March 2020, with suspicion of COVID-19 infection. The patient was complaining of labor pain prior to presentation.

The objective of this study is to report the case and to describe the challenges that are faced while dealing with a case of COVID-19 pregnant patient, during labor, delivery, and surgical intervention. This case reports a patient in labor pain with suspicion of COVID-19 infection due to contact with a positive COVID-19 family member. With no clinical signs or symptoms consistent with the disease, and positive polymerase chain reaction (PCR) outcome for COVID-19 later on, the hospital main departments conducted an active contact tracing and reviewed the preparation and infection prevention control precautions.

The most common problem with COVID-19 is the low level of awareness between healthcare workers related to infection prevention and transmission of the COVID-19 virus. The illness can be better handled and the medical team can be more secure by enhancing the education, case triage, proper guideline and protocols to be implemented appropriately.

## Introduction

Since December 2019, the epidemic of coronavirus infection COVID-19, which started in Wuhan, China, has come to be a global community wellbeing threat [1]. In February 2020, the spread of COVID-19 was considered as very high risk at the global level. World Health Organization (WHO) upgraded its assessment accordingly. COVID-19 was described as pandemic in March 2020. In the same month, Saudi Arabia reported confirmed cases of COVID-19 patients coming from abroad [2]. By May 20, 2020, around 4.7 million patients have been stated worldwide, causing more than 318,000 deaths. The pandemic has spread to 118 countries all over the world [3].

One of the most common challenges with COVID-19 is the lack of a full understanding of infection prevention and transmission among healthcare personnel. As stated in the reports of the healthcare-related COVID-19 spread in China, the majority of the incidents are due to the health care staff’s lack of awareness about the avoidance and control of infectious diseases [4]. Health staff expected to go into close bodily contact with patients should strictly follow the standardized practices to go into and leave infected places [5].

Pregnant women are considered vulnerable due to the physiological adaptive changes during this period, and they could be further prone to have COVID-19 than the overall population. As COVID-19 pandemic is fast growing, caring of pregnant women and fetal security has become a main concern; however, there are a little evidence on evaluation and managing of pregnant women infected with COVID-19, and the possible hazards of spread of infection from the mother to the fetus is ambiguous [6].

The obstetrical population have various contacts with the health care organization and the majority are admitted to the health care settings for labor and delivery, so they are presenting a unique challenge during COVID-19 pandemic [7]. According to our literature review, several clinical serious outcomes happened with women during gestational period with COVID-19 [8].

At this point, we report a patient who, in prenatal period, diagnosed with COVID-19, and mercifully, delivered a healthy newborn. This article focuses on all the details from admission till the postpartum period. The other aim of this study was to describe the challenges that were faced while dealing with a case of COVID-19 pregnant patient - during labor, delivery, and surgical intervention in an era of limited data available about the disease.

## Case presentation

A 38-year-old Saudi female patient - gravida 4 para 2+1, 38 weeks of gestation - presented to our hospital on 27th of March 2020 in obstetrics and gynecology emergency with labor pain and history of contact with COVID-19 positive patient who had diagnosed one day back (her brother) with no respiratory or gastrointestinal symptoms related to COVID-19. The patient declared no speciﬁc medical disease and had no issue in her medication and family history (her occupation: housewife).

She got score 5 out of 8 in respiratory triage, and isolated as she was stable case. The patient stayed in the droplet, contact, and standard precaution in negative pressure room, and all health care workers were informed by label on the isolation room door.

**Figure 1:**
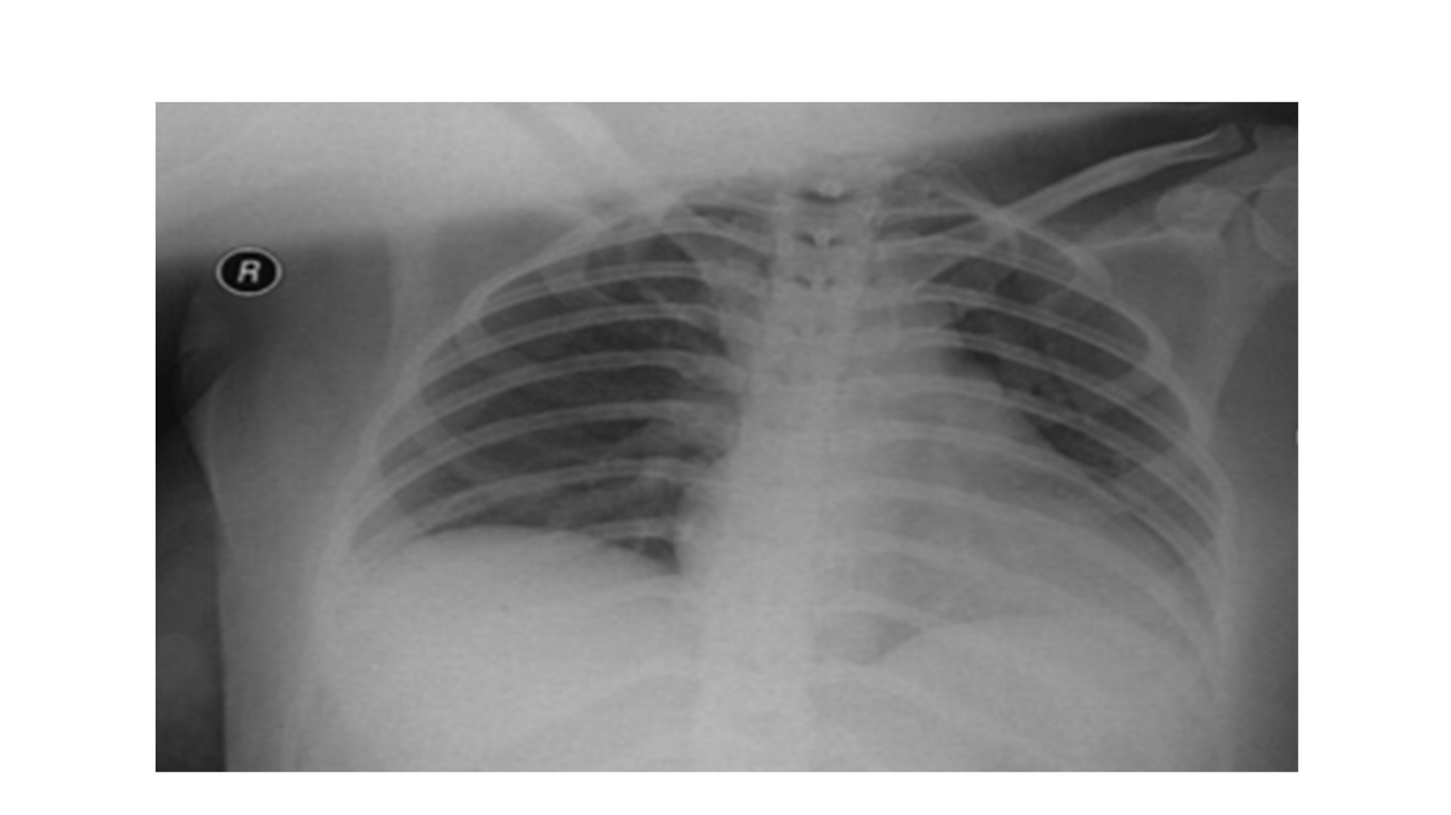
Chest X-ray.

All the prenatal period scheduled screening examinations and tests were completed. In addition, all of these were within normal range. Her clinical examinations showed temperature (T) 37.2 °C, heart rate (HR) 87/min, respiratory rate (RR) 20/min, blood pressure (BP) 110/70 mmHg, O2 saturation 97%, and fetal heart rate (FHR) 170/min. Laboratory examination was within the normal range (Table 1), and chest X-ray was normal (Figure 1).

**Table 1:** Laboratory results of the patient during hospitalization. WBC: White blood cell; RBC: Red blood cell; Hgb: Hemoglobin; HCT: Hematocrit; MCV: Mean corpuscular volume; MCH: Mean corpuscular hemoglobin; MCHC: Mean corpuscular hemoglobin concentration; PLT: Platelet; RDW-SD: Red blood cell distribution width-standard deviation; RDW-CV: Red blood cell distribution width-coefficient of variation; MPV: Mean platelet volume; Glu: Glucose; CREA: Creatinine; TBIL: Total bilirubin; AST: Aspartate aminotransferase; ALT: Alanine transaminase; Na: Sodium; K: Potassium; Cl: Chlorine; PT-THS: Prothrombin time; APTT-PSL: Partial thromboplastin time; INR: International normalized ratio.

Test	Result	Unit
WBC	8.34	10^3/uL
RBC	4.62	10^6/uL
HGB	12.1	g/dL
HCT	28.1	%
MCV	82.5	fL
MCH	26.2	pg
MCHC	31.8	g/dL
PLT	258	10^3/uL
RDW-SD	48.0	fL
RDW-CV	15.9	%
RDW	13.3	fL
MPV	11.5	fL
GLU	116.1	mg/dL
Urea	1.8	mmol/L
CREA	49	umol/L
TBIL	0.372	mg/dL
AST	17.4	U/L
ALT	8.9	U/L
Na	133	mmol/L
K	4.33	mmol/L
Cl	100.4	mmol/L
PT-THS	11.4	sec
APTT-PSL	27.8	sec
INR Blood Group	0.99 O +ve	

An urgent meeting was held involving: Infection Preventing and Control, Medical Services, Nursing Office, Operation Room, Anesthesia, Obstetrics and Gynecology, and Neonatal Departments.

We assigned a well-trained team from concerned departments to handle the patient, the pathway from the emergency department to the operating room was cleared, the patient was transferred with all precautions to the assigned operation room supplied by high-efficiency particulate air filter (HEPA filter) and all scheduled surgeries were in hold at the time of cesarean section operation. All healthcare providers followed the personal protective equipment (PPE) recommendations.

Cesarean section was done smoothly under spinal anesthesia with no specific complications, the newborn received and assessed by neonatologist with normal evaluation. Then the neonate was shifted to the neonatal care unit for isolation after routine care and the mother was recovered in the same operating room. After recovery, she was shifted to the isolation ward. The patient received pre- and post-surgery antibiotic surgical prophylaxis as per guideline. Terminal cleaning and fumigation were done for the assigned operation room immediately. As per the Saudi Ministry of Health protocol, we continued her isolation and swabbing every three days, and she was transferred after two weeks to quarantine.

Table 2 shows that in this situation 29 of health care workers were exposed to the patient and the procedure while wearing a surgical mask, or N95 masks. Even with all of the infection control procedure one of the health care workers in this situation acquired infection.

**Table 2:** Health workers who were in contact with the patient.

			Total
Contact by job categories			
	Physicians	7	29
	Nurses	17	
	Anesthesia Technician	1	
	Receptions	2	
	Housekeepers	2	
Contact by risk level.			
Low Risk	Physician	4	21
	Nurse	13	
	Anesthesia Technician	1	
	Reception	1	
	Housekeeper	2	
High Risk	Physician	3	8
	Nurse	4	
	Reception	1	
Nasopharyngeal swabbing result.			
Positive Result	1 (high-risk group)		

Contact tracing for all health care workers exposed to the patient was undertaken upon receiving a positive nasopharyngeal swab result after two days from operation.

## Discussion

There are few cases of pregnancy with COVID-19 reported in the literature, and even fewer reported during labor. The initial approach of COVID-19 treatment during pregnancy does not differ greatly from the non-pregnant patient. However, it presents some peculiarities.

There are small number of clinical drug experiments that were performed during antenatal period. Along with the unique condition of pregnancy, we collected many indications for drug choice for efficiency and safety treatment in case of pregnancy with COVID-19, which included chloroquine and its analogues, drugs of high dose therapy (HDT) (Metformin, statins), lobinavir/ritonavir, glycyrrhizic acid, and nanoparticle mediated drug delivery (NMDD) [9].

The case was a 38-year-old female in her 38 weeks of pregnancy with no COVID-19 symptoms. In the diagnostic tests, she had slightly leukocytosis. The patient endured a C-section. The baby had no health threat condition and considered a well baby. This result seems in another study done in city of New York which reported that majority of the positive for SARS-CoV-2 cases at delivery were asymptomatic, and near to twelve percent of asymptomatic cases who were entered to labor ward were positive for SARS-CoV-2 [7].

Another study assessed the consequence of COVID-19 on nine pregnant cases without any diseases (all gestational ages ≥36 weeks). The symptoms of pregnant cases with COVID-19 pneumonia were varied, with the major symptoms being fever and cough. No intrauterine fetal death, neonatal asphyxia, still-birth, or parental death was detected. Nevertheless, in neonates, 44% and 22% were premature birth and had low birth-weight, respectively [10].

The present study showed that the patient delivered by cesarean section. It was in consistence with Liu et al. study which evaluated the pregnant cases suffered from COVID-19 and found that 77% undertaken cesarean section due to numerous causes involving premature rupture of the membrane, fetal distress, and death of a fetus. Also, premature birth was detected in 46% of all cases [11].

Related to the treatment plan, in our case there was no specific plan. It's in accordance with another study which reported that there is no specific drugs for a symptomatic patient [12].

In our case, separation of the neonate from the mother was done. It seems in the same line with another studies which found that cautionary separation between neonates and mothers is a challenge in obstetric infection control, with inadequate data to guide care plan suggested by different countries [13].

In our case, cesarean section was done smoothly under spinal anesthesia with no specific complications. As reported in Wang et al. study, intubating cases with COVID-19 is a high-risk technique which increases the exposure of the health care workers to airway discharges, which can transmit a viral infections [14]. In accordance with our findings another study reported that cases without symptoms and cases with minor disease have less complications [15].

For infection control, in our case the staff in operating room used all the PPE supplies. Also, the number of infected personnel from health care workers was small and the staff were from the front-line staff. These results were in contrast with the study done by Hunter et al., who found that nonclinical staff had similar positivity rates of infection to front-line staff [16]. It's unlike a study of Ng et al., who reported that single PPE strategy is without problems or benefits [17].

Guidelines published by CDC reported that, surgical face masks and eye protection should be worn when working in close contact with patients with suspected COVID-19 or in any area where COVID-19 patients contacted [18].

## Conclusions

One of the most common problems with COVID-19 is the lack of full understanding among healthcare personnel related to infection prevention and transmission of the COVID-19 virus. The protection of health staff is essential despite the increased demand and global shortage of PPE. Availability of updated guidelines at this time can provide better way for dealing and handling with positive COVID-19 cases and consequently better outcome. The current available Saudi Ministry of Health (MOH) guidelines representing the most updated evidence-based practice related to COVID-19 may provide better care for patients and health care workers' safety. Also, the study concludes that current isolation protocols and personal protective equipment appear sufficient to prevent high levels of nosocomial transmission to frontline staff in our setting.
